# Transcriptome sequencing reveals novel molecular features of SLE severity

**DOI:** 10.3389/fgene.2023.1121359

**Published:** 2023-07-24

**Authors:** Xiaojing Zhang, Jiali Zhang, Zhaobing Pan, Yuxi Zhang, Xiaoqing Xu, Yujun Sheng, Zhengwei Zhu, Fusheng Zhou, Leilei Wen

**Affiliations:** ^1^ Department of Dermatology, The First Affiliated Hospital, Anhui Medical University, Hefei, Anhui, China; ^2^ Institute of Dermatology, Anhui Medical University, Hefei, Anhui, China; ^3^ Key Laboratory of Dermatology (Anhui Medical University), Ministry of Education, Hefei, Anhui, China; ^4^ Inflammation and Immune Mediated Diseases Laboratory of Anhui Province, Hefei, Anhui, China

**Keywords:** Autoimmune Diseases, Systemic Lupus Erythematosus (SLE), RNA-seq, Treg, scRNA-seq, AMPH

## Abstract

**Introduction:** Systemic lupus erythematosus (SLE) is an autoimmune disorder characterized by the production of autoantibodies, immune complex deposition, and tissue/organ damage. In this study, we aimed to identify molecular features and signaling pathways associated with SLE severity using RNA sequencing (RNA-seq), single-cell RNA sequencing (scRNA-seq), and clinical parameters.

**Methods:** We analyzed transcriptome profiles of 45 SLE patients, grouped into mild (mSLE, SLEDAI ≤ 9) and severe (sSLE, SLEDAI > 9) based on SLE Disease Activity Index (SLEDAI) scores. We also collected clinical data on anti-dsDNA, ANA, ESR, CRP, snRNP, AHA, and anti-Smith antibody status for each patient.

**Results:** By comparing gene expression across groups, we identified 12 differentially expressed genes (DEGs), including 7 upregulated (CEACAM6, UCHL1, ARFGEF3, AMPH, SERPINB10, TACSTD2, and OTX1) and 5 downregulated (SORBS2, TRIM64B, SORCS3, DRAXIN, and PCDHGA10) DEGs in sSLE compared to mSLE. Furthermore, using the CIBERSORT algorithm, we found that Treg cells were significantly decreased in sSLE and negatively correlated with AMPH expression, which was mainly expressed in Treg cells from SLE patients according to public scRNA-seq data (GSE135779).

**Discussion:** Overall, our findings shed light on the molecular mechanisms underlying SLE severity and provide insight into potential therapeutic targets.

## 1 Introduction

SLE is a disease of the immune system caused by excessive activation of immune cells and secretion of large amounts of autoantibodies (Tsokos, 2011). The dysregulation of innate and adaptive immunity leads to the occurrence of diseases. It has been reported that the imbalance of T cells, B cells, and dendritic cells are related to the pathogenesis (Li et al., 2021), especially CD4^+^ T cells imbalance (Yuliasih et al., 2019). The imbalance of the Th17/Treg ratio affects the occurrence and development of SLE inflammation (Pan et al., 2020). And aggravates organ damage, so the mechanism of regulatory T cells (Treg) defect is constantly explored. For example, OX40L/OX40 axis leads to Foxp3+ T cells decline (Jacquemin et al., 2018), Oxidative stress activates mTORC1 and mTORC2 pathways and inhibits Treg development. A low dose of IL-2 increased the number of functional and thymus-derived Foxp3+ T cell populations (Scheinecker et al., 2020; Grasshoff et al., 2021). Inhibition of glycolysis, lipid metabolism, and mTOR signaling can reverse Th17/Treg imbalance (Shan et al., 2020). Direct injection of Treg cells into lupus mice controlled inflammatory responses and improved pathological lesions (Scalapino and Daikh, 2009). These studies have fully demonstrated that enhancing the number and function of Treg cells is beneficial for SLE patients (Yang et al., 2011). However, the role of Treg in contributing to the severity of SLE is not well understood.

In order to investigate the pathogenesis of SLE, many high-throughput sequencing studies have been conducted in recent years. For example, Nikolaos I Panousis et al. (Panousis et al., 2019) conducted transcriptome sequencing and gene analysis of SLE blood mRNAs and found targeted sites for SLE susceptibility and severity. Evan et al. (Der et al., 2017) identified T cell populations in the renal tissue of lupus kidneys by sequencing. In a cohort study of 136 lupus patients and 89 healthy controls in an East Asian population, Nakano et al. (2022) investigated the molecular characteristics of immune cells in terms of disease status and disease activity and revealed that disease activity had the greatest impact on clinical heterogeneity. Here, we explored the transcriptome characteristics of severe SLE in a Chinese population using high-throughput sequencing technology.

Here, advanced transcriptome technology is applied to explore the pathogenesis of SLE, and genes regulating the severity of SLE are found, as well as changes in the severity of the disease that will affect the distribution of Treg in SLE.

## 2 Materials and Methods

### 2.1 Sample collection

A total of 45 female SLE patients (*n* = 45, mean age 43.2 ± 13.85 years) were included in this study, all of whom were from the Department of Rheumatology and Immunology of the First Affiliated Hospital of Anhui Medical University from February 2021 to May 2022. All SLE subjects met at least four of SLE classification criteria of the American College of Rheumatology (ACR) (Font and Cervera, 1993). Disease activity was assessed using the SLE Disease Activity Index (SLEDAI) (Liang et al., 1989). The SLEDAI score was used to classify patients into mild SLE (mSLE, SLEDAI≤9) and severe SLE (sSLE, SLEDAI >9). To exclude confounding factors, blood was collected from all patients on the second day of hospital admission, before receiving any treatment. There were no other diseases such as infection, cancer, or pregnancy. All patients had been off medication for more than 1 year or had not taken any medication. The study was approved by the Ethics Committee of the First Affiliated Hospital of Anhui Medical University, and all subjects provided written informed consent. Clinical parameters of the patients are shown in Supplementary Table S1. Whole blood samples (3–10 mL) were collected from each patient, and PBMCs were extracted from each blood sample according to the standard centrifugation method and sent to BGI Genomics Company.

### 2.2 PBMC and RNA isolation

A total of 6 mL peripheral blood was collected from each sample, and peripheral blood mononuclear cells (PBMCs) were isolated using density gradient centrifugation with Ficoll-Paque plus (17144003, Cytiva). After washing twice in PBS, PBMCs were preserved in serum-free cell lyophilization solution (abs9417, Absin) and were stored in liquid nitrogen at −196°C until thawing, and sent to BGI Genomics Company. Total RNA was extracted from cells using TriZol Reagent (15596026, Thermo Fisher Scientific) according to the manufacturer’s instructions. RNA quality control was conducted with a NanoDrop spectrophotometer and an Agilent 2100 Bioanalyzer (Thermo Fisher Scientific). Isolated RNA was stored at −80°C for use.

### 2.3 Sequencing platform and library preparation

The library was prepared according to DNBSEQ platform process. mRNA molecules were purified from total RNA using oligo (dT)-attached magnetic beads. The purified mRNA molecules were fragmented into small pieces using fragmentation reagent after a certain period of reaction at an appropriate temperature. First-strand cDNA was generated using random hexamer-primed reverse transcription, followed by a second-strand cDNA synthesis. The synthesized cDNA was subjected to end-repair and then was 3′ adenylated. Adapters were ligated to the ends of these 3′ adenylated cDNA fragments, and the resulting cDNA fragments were amplified with adapters from previous step. PCR products were purified with Ampure XP Beads (AGENCOURT) and dissolved in EB solution. The library was validated on the Agilent Technologies 2100 bioanalyzer. The double stranded PCR products were heat-denatured and circularized by the splint oligo sequence. The single-strand circular DNA (ssCir DNA) was formatted as the final library. The library was amplified with phi29 to make DNA nanoballs (DNBs), each of which contained more than 300 copies of one molecular. The DNBs were load into the patterned nanoarray, and single end 50 (pair-end 100/150) base reads were generated using the combinatorial Probe-Anchor Synthesis (cPAS) method. The qualified RNA samples were measured using the DNBSEQ platform from BGI Genomics Company, and each sample yielded an average of 6.67 Gb of raw sequencing data. When the samples were mapped to the human reference genome hg19, the average matching rate was 94.50%, and the average matching rate for the mapped gene set was 66.29%. A total of 17,826 genes were detected.

### 2.4 Differentially expressed genes screening

Cufflinks software calculated gene expression values in fragments per kb per million (FPKM). Dimension reduction and visualization of data were generated using principal component analysis (PCA). Differentially expressed genes (DEGs) were identified by the R package “limma” (version: 3.40.2) and were visualized in volcano plots. An adjusted *p* value of < 0.05 and a |log2FC| ≥ 1 were considered to indicate a statistically and biologically significant difference. The violin plot represents the gene sample according to log10 (gene expression). A thick line (black) within the box indicates the mean.

### 2.5 KEGG and GSEA enrichment analyses

Kyoto Encyclopedia of Genes and Genomes (KEGG) enrichment analyses of the upregulated and downregulated DEGs were performed using the R ClusterProfile package. A cutoff criterion of *p* < 0.05 was used to indicate significant differences in the KEGG pathways. The present study reports the KEGG pathways. To explore the biological signaling pathway, hallmark gene set enrichment analysis was performed using GSEA (v.4.1.0) (Subramanian et al., 2005). KEGG pathways with significant enrichment results were determined based on net enrichment score (NES), gene ratio, and *p* value. Gene sets with |NES| > 1, NOM *p* < 0.05, and FDR q < 0.25 were considered to be significantly enriched.

### 2.6 Screening clinically relevant DEGs

Based on the patient’s clinical information, we screened DEGs for six clinical indicators that are highly variable in SLE, namely, ESR, CRP, SSA52, AHA (Marder, 2019), snRNP(Vollmer et al., 2005; Marshak-Rothstein, 2006), and anti-Smith (Ahn et al., 2019). UpSet bar charts were constructed using UpSetR (Conway et al., 2017). The correlation between upregulated and downregulated DEGs and SLEDAI was analyzed by Spearman’s correlation coefficient and plotted using the R package “corrplot.” Protein‒protein interaction (PPI) networks, assayed by STRING (http://string-db.org/), were constructed using the top 10 scoring proteins for further exploration of gene relationships in immune-related pathways and imported into Cytoscape software for visualization.

### 2.7 Immune cell composition

The CIBERSORT algorithm (Newman et al., 2015) was used to calculate the proportions of various immune cells for each patient. The heatmap displays the proportion of 10 types of immune cells in all samples. The R package “ggpubr” was used to visualize the proportions of 10 types of immune cells in the mSLE and sSLE groups. T-test statistics were used to evaluate the proportion differences between groups, with *p* < 0.05 as a significant difference.

### 2.8 scRNA sequencing data mining

We retrieved the public single cell RNA sequencing (scRNA-seq) dataset GSE135779, which consisted of 33 children with SLE and 11 child normal controls. The Seurat (Korsunsky et al., 2019) format data were merged by the hormony algorithm. After filtering cells with a percentage of mitochondrial DNA >10% and featured RNA <200 or >4000, we finally obtained 280,529 cells. We then used Seurat to identify highly variable genes, dimensionality reduction, and standard unsupervised clustering algorithms and determine the cell identity using the marker gene list in the published Nature Immunology (Nehar-Belaid et al., 2020). T cell subsets were extracted to examine the expression of the above relevant genes.

## 3 Results

### 3.1 Identifying DEGs in severe and mild SLE

**FIGURE 1:**
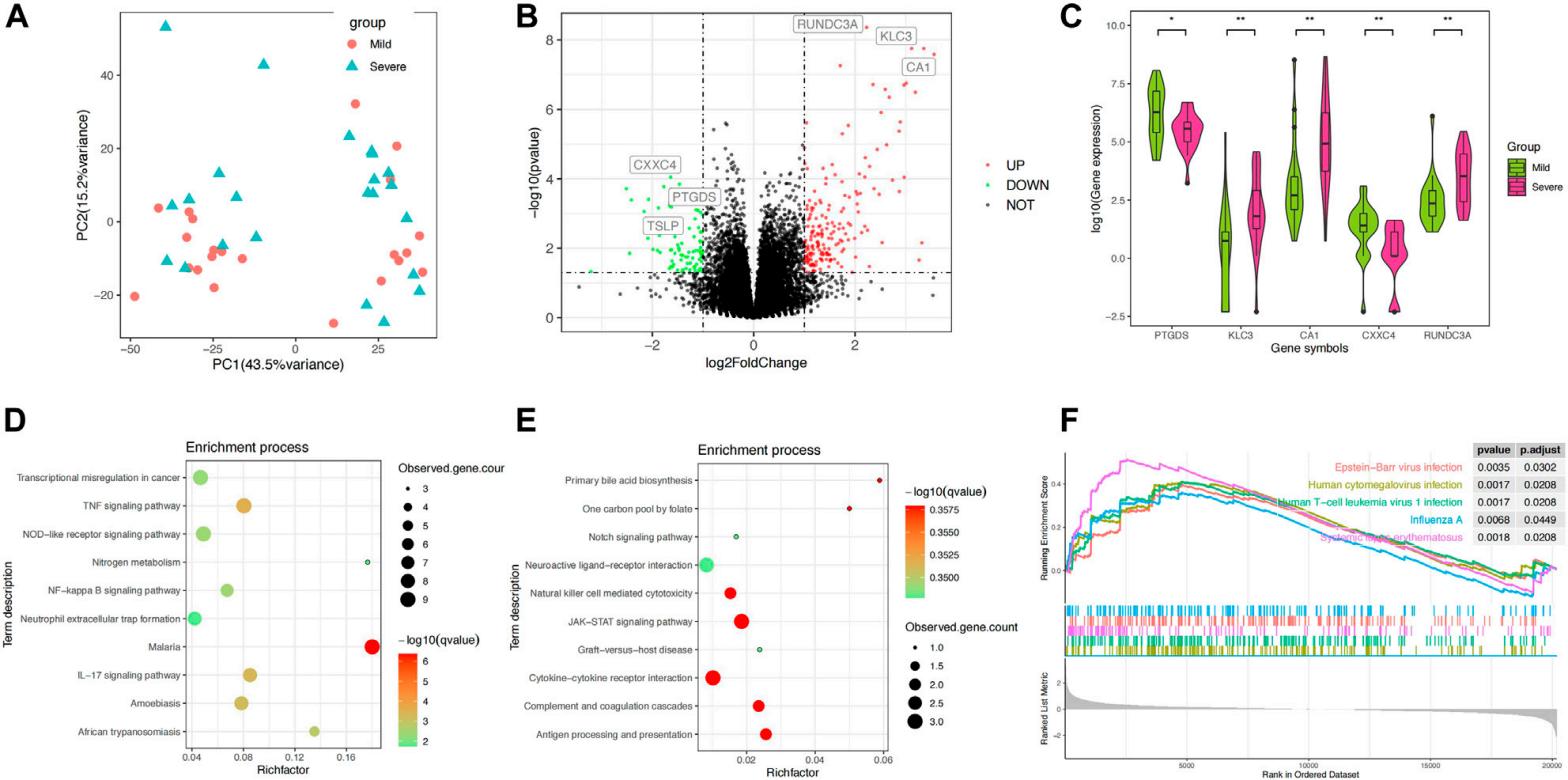
Differential analysis of the blood transcriptomes between mSLE and sSLE. **(A)** PCA showed the characteristics of gene expression levels in 45 samples. Each dot indicates a sample. The red dot represents mSLE, whereas the blue dot represents sSLE. The proportion of variance explained is indicated in parentheses. **(B)** Volcano plot describing the distribution of downregulated and upregulated DEGs. Green and red represent lower and higher expression of genes in the corresponding group, respectively. X-axis: fold change; Y-axis: −log10 *p* value. **(C)** Violin plot analysis comparing the distribution of the most significantly upregulated and downregulated five DEGs in sSLE and mSLE. **(D,E)** KEGG enrichment analysis of upregulated DEGs **(D)** and downregulated DEGs **(E)**. KEGG pathway enrichment analysis of key targets (top 10 are listed); the abscissa label represents the rich factor of pathways. The colors of the dots represent the *p* values of enrichment, and the size of the dots represents the number of enriched genes. **(F)** Hallmark gene set enrichment analysis (GSEA) showing pathways enriched in sSLE compared to mSLE. NES: normalized enrichment score; NOM *p* value: nominal *p* value; FDR q-value: false discovery rate.

To investigate whether altered gene expression leads to the severity of SLE, all patients were divided into mild SLE (mSLE, SLEDAI≤9) and severe SLE (sSLE, SLEDAI>9) groups according to the SLEDAI score. PCA (Figure 1A) showed that the patients in the mSLE and sSLE groups were scattered and not completely distinguishable, reflecting the heterogeneity of SLE, in agreement with previous studies (Panousis et al., 2019).

Using the filtering criteria, we identified 180 upregulated DEGs and 83 downregulated DEGs in sSLE (Figure 1B). Among them, RUNDC3A (*p* = 4.37E−9, log2FC = 2.22), KLC3 (*p* = 1.77E−8, log2FC = 3.11) and CA1 (*p* = 2.60E−8, log2FC = 3.55) were the most upregulated, and CXXC4 (*p* = 8.95E−5, log2FC = −1.63), PTGDS (*p* = 1.43E−4, log2FC = −1.46) and TSLP (*p* = 5.16E−3, log2FC = −2.08) were the most downregulated (Table 1; Figure 1C). KEGG analysis revealed that the upregulated DEGs were significantly enriched in the TNF signaling pathway, IL-17 signaling pathway, NF-κB signaling pathway, neutrophil extracellular trap formation, NOD-like receptor signaling pathway, and other immune inflammation-related signaling pathways (Figure 1D). Downregulated DEGs were significantly enriched in the JAK-STAT signaling pathway, natural killer cell-mediated cytotoxicity, cytokine‒cytokine receptor interaction, complement, coagulation cascades, and antigen processing and presentation (Figure 1E). We also performed GSEA enrichment analysis, which showed that upregulated DEGs were significantly enriched in four pathways, including Epstein‒Barr virus infection, human cytomegalovirus infection, human T cell leukemia virus 1 infection, and influenza A infection (Figure 1F).

**TABLE 1:** Detailed information of 20 top DEGs in sSLE group.

Gene name	Official full name	Chr*	Log2FC#	Padjust(<0.05)	Trend
SERPINB1	serpin family B member 1	6	1.47	7.77 × 10^−4^	Up
AMPH	Amphiphysin	7	1.77	1.06 × 10^−2^	Up
RUNDC3A	RUN domain containing 3A	17	2.22	4.37 × 10^−9^	Up
HBA1	hemoglobin subunit alpha 1	16	2.59	2.66 × 10^−7^	Up
IFIT1B	interferon induced protein with tetratricopeptide repeats 1B	10	2.96	2 × 10^−7^	Up
HBM	hemoglobin subunit mu	16	3.01	1.75 × 10^−7^	Up
KLC3	kinesin light chain 3	19	3.11	1.77 × 10^−8^	Up
AHSP	alpha hemoglobin stabilizing protein	16	3.35	1.77 × 10^−8^	Up
GYPA	glycophorin A (MNS blood group)	4	3.35	4.54 × 10^−8^	Up
CA1	carbonic anhydrase 1	8	3.55	2.6 × 10^−8^	Up
RSPH6A	radial spoke head 6 homolog A	19	−2.51	1.92 × 10^−4^	Down
PRSS35	serine protease 35	6	−2.41	4.04 × 10^−4^	Down
TSLP	thymic stromal lymphopoietin	5	−2.08	5.16 × 10^−3^	Down
CLEC4F	C-type lectin domain family 4 member F	2	−2.07	3.82 × 10^−4^	Down
S100B	S100 calcium binding protein B	21	−1.77	1.68 × 10^−4^	Down
NFIB	nuclear factor I B	9	−1.66	5.84 × 10^−4^	Down
CXXC4	CXXC finger protein 4	4	−1.63	8.95 × 10^−5^	Down
C19orf33	chromosome 19 open reading frame 33	19	−1.62	6.54 × 10^−4^	Down
PTGDS	prostaglandin D2 synthase	9	−1.46	1.43 × 10^−4^	Down
LGALS9B	galectin 9B	17	−1.41	4.16 × 10^−4^	Down

Chr*: Chromosome; Log2FC#: |log2 fold change| ≥ 1; Padjust: The value after correction for the significance P value.

### 3.2 Identifying SLE phenotype-associated DEGs

**FIGURE 2:**
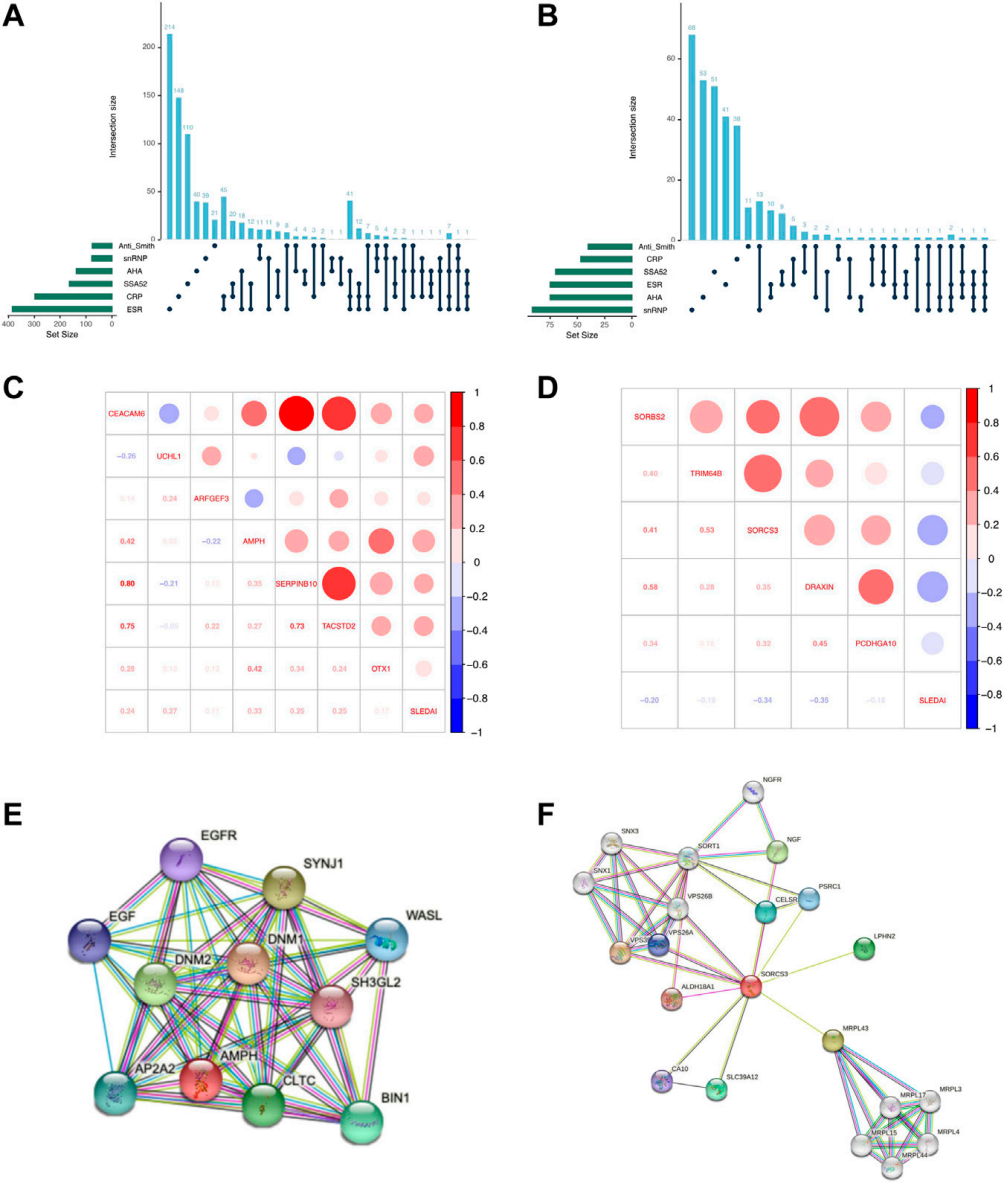
Analysis of clinical indicator-related differentially expressed genes. **(A,B)** Upset plot indicates overlapping genes between ESR, CRP, SSA52, AHA, snRNP, and anti-Smith. DEGs shared between the six phenotypes are indicated by linked dots below the x-axis. The top number above each bar represents the number of DEGs that are unique to each group. **(C,D)** Heatmap of Spearman’s correlation coefficients of SLEDAI and upregulated **(C)** and downregulated **(D)** genes. Red represents a positive correlation, and blue represents a negative correlation. **(E,F)** PPI network of AMPH **(E)** and SORCS3 **(F)**. Circles represent genes, and lines represent interactions between genes.

To further investigate the correlation between expression levels and disease severity, we divided 45 SLE patients into phenotype-positive and phenotype-negative groups according to the clinical parameters ESR, CRP, SSA52, AHA, snRNP, and anti-Smith antibody. Differentially expressed genes for six statuses were classified into upregulated and downregulated DEGs. We used the “Upset R” package to find the common and overlapping genes across the six groups. As shown in Figures 2A, B, there were some overlaps between the different clinical phenotypes. We found that a large percentage of DEGs, 70.9% (572) of upregulated DEGs and 78.2% (251) of downregulated DEGs, only linked with one phenotype (Figures 2A, B). In addition, 1.1% (9) of upregulated DEGs and only 1.6% (5) of downregulated DEGs were observed in four phenotypes (Figures 2A, B). Among these genes, we focused on seven upregulated DEGs (CEACAM6, UCHL1, ARFGEF3, AMPH, SERPINB10, TACSTD2 and OTX1) and 5 downregulated DEGs (SORBS2, TRIM64B, SORCS3, DRAXIN and PCDHGA10).

Correlation analysis showed that all 7 upregulated genes were positively correlated with SLEDAI score (Figure 2C), with the largest Spearman’s correlation coefficient of 0.33 for AMPH. AMPH showed upregulation in SLE patients with SSA52 (*p* = 7.8E-3, log2FC = 2.58), AHA (*p* = 3.1E−3, log2FC = 2.02), ESR (*p* = 1.4E-4, log2FC = 3.46), and CRP (*p* = 6.3E-4, log2FC = 2.86). All 5 downregulated genes were negatively correlated with SLEDAI score (Figure 2D), with the highest Spearman’s correlation coefficient of −0.34 for SORCS3. SORCS3 showed downregulation in SLE patients with ESR (*p* = 4.5E−3, log2FC = −1.57), snRNP (*p* = 2.2E−4, log2FC = −2.10), AHA (*p* = 4.8E−4, log2FC = −1.77), and anti-Smith (*p* = 8.6E−3, log2FC = −1.53). A PPI network, using the web version of STRING, showed that AMPH was linked with BIN1, EGF/EGFR, and CLTC (Figure 2E) and that SORCS3 was linked with NGF, SLC39A12 and VPS26A (Figure 2F), some of which have been implicated in SLE etiology.

### 3.3 Relationship between the severity of SLE and tregs

**FIGURE 3:**
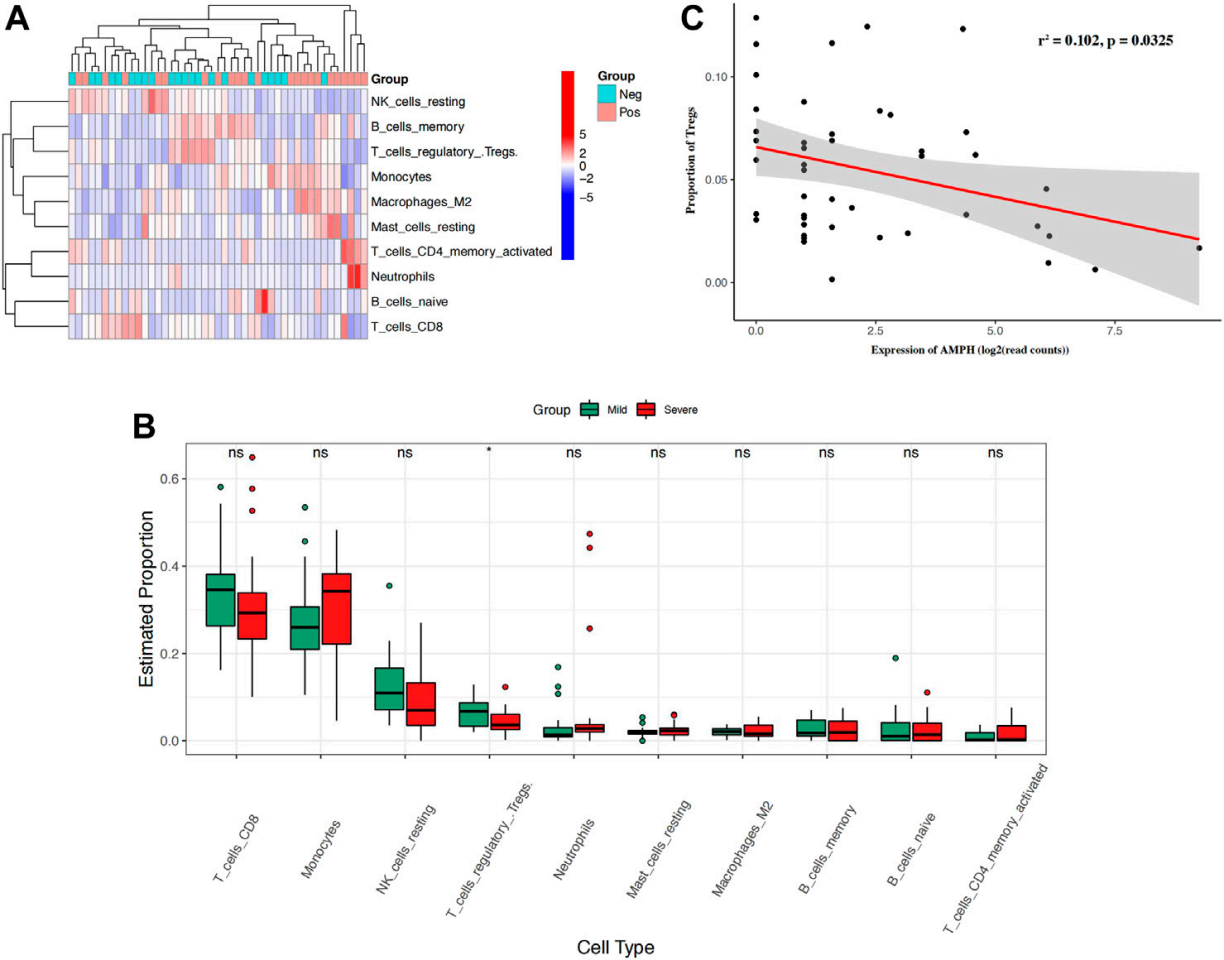
The proportion of immune cells in SLE patients and the correlation between the expression of AMPH and the proportion of Tregs. **(A)** The heatmap shows the proportion of 10 types of immune cells in 43 SLE patients. **(B)** The box plot shows the difference in the proportion of 10 immune cell types in mSLE and sSLE. **(C)** Negative linear relationship between AMPH and Tregs. *p < 0.05 and **p < 0.01.

SLE patients usually present a disturbed proportion and aberrant function of a series of immune cells. T cell and B cell abnormalities have long been described in SLE and are thought to be central to the disease process. To reveal whether immune cells affect SLE severity, we introduced a CIBERSORT algorithm to infer the immune cell proportion. Based on the bulk transcriptome data, CIBERSORT was employed to calculate the proportions of 10 immune cell types in the peripheral blood of mSLE and sSLE patients (Figure 3A). The results showed that the proportions of Tregs in sSLE were significantly lower than those in mSLE (Figure 3B). Furthermore, AMPH expression was negatively correlated with the proportion of Tregs (Figure 3C). Compared with mSLE, monocytes, neutrophils, resting mast cells, M2 macrophages and memory-activated CD4^+^ T cells were slightly higher in sSLE, while CD8^+^ T cells and resting natural killer (NK) cells were lower, but they were not significant.

### 3.4 scRNA-seq validates AMPH-Treg correlation

**FIGURE 4:**
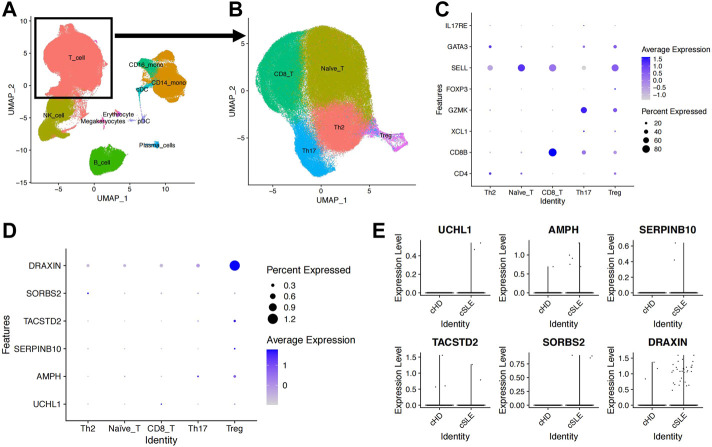
Upregulated AMPH in Treg cells from the GSE135779 dataset. **(A)** UMAP plot of the eight cell subpopulations. **(B)** UMAP plot of the five T-cell subpopulations: naive T cells (63,861), CD8+ T cells (36,609), Th2 (28,732), Th17 (14,845) and Treg (2,824). **(C)** The dot plot shows cell type-specific markers for each T-cell subset. **(D)** The dot plot indicates the expression level and percentage of expressed cells for the six target genes. **(E)** Expression levels of the six target genes between the SLE and control groups.

Through the analysis of the scRNA-seq dataset (GSE135779), we obtained transcriptomes of 280,259 cells and identified eight main subgroups, including T cells, monocytes, natural killing cells (NK cells), B cells, plasma cells, erythrocytes, megakaryocytes and dendritic cells (DCs), after removing doublets and correcting batch effects (Figure 4A). Specifically, the T cells were further classified into 63,861 naive T cells (SELL), 36,609 CD8^+^ T cells (CD8B), 28,732 T helper 2 cell (Th2, CD4, GATA3), 14,845 Th17 (IL17RE) and 2,824 regulatory T cells (Tregs, FOXP3) (Figures 4B, C). Taking advantage of the scRNA data, we focused on examining the expression levels of 12 genes associated with SLE severity in Treg cells. Among these genes, only four upregulated genes (AMPH, UCHL1, SERPINB10, and TASTD2) and two downregulated genes (DRAXIN and SORBS2) were observed. The failure to extract the expression levels of other genes might be attributed to the limited number of annotated Treg cells in this dataset (Figure 4D). When comparing the expression levels of these genes in the SLE groups and control groups, we consistently found that the expression levels of AMPH and SERPINB10 were upregulated in the SLE groups, indicating their potential involvement in Treg development (Figure 4E).

## 5 Discussion

SLE is a complex autoimmune disease that involves multiple immune cells. In this study, we compared the transcriptome profiles for the sSLE and mSLE groups and identified 180 upregulated DEGs and 83 downregulated DEGs in sSLE patients. KEGG and GSEA pathway enrichment analyses revealed that DEGs were significantly enriched in the TNF, NF−kappaB and IL−17 signaling pathways. By overlapping phenotype-specific DEGs, we identified seven upregulated DEGs (EACAM6, UCHL1, ARFGEF3, AMPH, SERPINB10, TACSTD2, and OTX1) and five downregulated DEGs (SOBS2, TRIM64B, SORCS3, DRAXIN and PCDHGA10). Among them, we found that AMPH was positively correlated with the SLEDAI and negatively correlated with the proportion of Tregs. scRNA-seq data verified that AMPH is involved in Treg development.

Measuring the severity of SLE is a critical way to evaluate the activity of SLE development, but the detailed mechanism remains largely unknown. Recently, several groups set out to find genetic markers or signaling pathways underlying this clinical feature. In a remarkable milestone, Masahiro et al. conducted a large-scale transcriptome study with 6,386 RNA sequencing data points covering 27 immune cell types from 136 SLE and 89 healthy donors. All immune cells were sorted from PBMCs according to specific cell markers. They comprehensively profiled cell type-specific transcriptomic features and observed several disease-state and disease-activity signatures, most of which reflected the characteristics of both disease establishment and exacerbation (Nakano et al., 2022). In contrast to Masahiro et al., we used bulk RNA sequencing from 45 PBMCs to evaluate the severity of SLE and further dissected the immune cell proportion using a mathematical strategy.

Our result showed that AMPH was linked with BIN1 (Deng et al., 2006), EGF/EGFR (Lee et al., 2015; Mejia-Vilet et al., 2021), and CLTC (Figure 2E), some of which have been implicated in SLE etiology. BIN1 and CLTC were both associated with antigen processing rapture in SLE patients (Armstrong et al., 2014; Xie et al., 2022). EGFR (Xie et al., 2023) is expressed in lupus kidney and can be used as a target for drug therapy, and EGF (Ngamjanyaporn et al., 2022) is an indicator to assess the effectiveness of lupus kidney treatment. We have highlighted AMPH in our study due to several lines of evidence that suggest its fundamental role in SLE. **Firstly**, AMPH, also known as AMPH-1, encodes a protein that is associated with the cytoplasmic surface of synaptic vesicles. Breast cancer has been linked to decreased AMPH-1 expression and activation of EMT and ERK pathways (Chen et al., 2018). Similarly, knockdown of AMPH-1 has been shown to lead to abnormal cell proliferation, which is also associated with activation of the Ras-Raf-MEK-ERK signaling pathway (Yang et al., 2019). Autoimmune diseases and cancer are often related, and some autoimmune diseasesshare similar inflammatory pathways with cancer (Elkoshi, 2022). SLE patients are known to be at risk of hematological or nonhematological tumors, such as leukemia, lung cancer and liver cancer (Masetti et al., 2021). It is speculated that this related pathway is also pathogenic in SLE, but further experiments are needed to verify it.

**Secondly**, we observed that AMPH was upregulated in SLE patients. Both our bulk RNAseq and Masahiro et al. datasets from Treg cells showed that AMPH was upregulated in the sSLE group. Most importantly, AMPH also showed increased expression in patients with SSA52, AHA, ESR and CRP but not in patients with snRNP and anti-Smith, suggesting that AMPH is most likely involved in promoting some but not all phenotypes. **Thirdly**, we found that AMPH was commonly expressed in CD4^+^ T cells and significantly upregulated in Treg. scRNA sequencing showed that the expression level of the AMPH in SLE was higher than that in the healthy control group. AMPH and Tregs showed a negative linear correlation, and the expression level of the AMPH in Tregs was also low, which was consistent with the positive correlation between AMPH and the SLEDAI. **Finally**, rTregs belong to a subset of CD4^+^ T cells that inhibit T cell immunity to avoid damage caused by excessive T cell activation (Cai et al., 2019). In tumor cells, activation of the MEK/ERK pathway increases the levels of serum interleukin-10 (IL-10) and transforming growth factor-β (TGF-β), which is key for Treg conversion (Zdanov et al., 2016; Cheng et al., 2019). Based on the upregulated AMPH in Tregs and the negative correlation between AMPH and the Treg proportion, we suspected that AMPH may be important for Treg proliferation and differentiation though the MEK/ERK pathway.

Another interesting marker, SORCS3, which was negatively associated with the SLEDAI, was significantly downregulated in the sSLE group. SORCS3 is a member of the vacuolar protein sorting 10 protein (VPS10p) receptor family and uses the NGF/p75NTR pathway in glioblastoma (GBM) to suppress cell invasion and proliferation (Zhang et al., 2022). Transcriptomic sequencing in patients with refractory diffuse large B cell lymphoma (DLBCL) showed increased expression of SORCS3 (Park et al., 2016). In addition, genetic analysis of the isolated Faroe Islands revealed SORCS3 as a potential multiple sclerosis risk gene (Binzer et al., 2016). SORCS3 is associated with both autoimmunity and tumors, but the specific pathway regulating SLE pathogenesis, perhaps NGF/p75NTR or other pathways, needs to be further explored.

Our immune infiltration analysis revealed that only Treg cells were significantly lower in sSLE than in mSLE. Although the pathogenesis of SLE has not been fully clarified, recent studies suggest that the disruption of CD4^+^ T cell immune homeostasis triggers autoimmune disorders and is one of the important mechanisms triggering the development of SLE (Ma et al., 2010; Lai et al., 2013). Th17 and Treg cells are two important immune regulatory cells developed by CD4^+^ T cells. Studies have demonstrated that Th17/Treg cell imbalance is closely related to the pathogenesis and disease activity of SLE (Kleczynska et al., 2011), and further experiments revealed that the reduction in Treg cell number or diminished function is the principal factor of their imbalance (Bonelli et al., 2008; Pan et al., 2012). Reports of Treg numbers are controversial, with most studies showing a decrease in the proportion of Tregs in patients with SLE (Zhao et al., 2019; Li et al., 2020; Wiesik-Szewczyk et al., 2021), while other studies have shown that the number of Tregs did not change or increase in patients with SLE (Jacquemin et al., 2018; Ferreira et al., 2019; Hanaoka et al., 2020). A recent meta-analysis suggested that the proportions of Treg/PBMC and Treg/CD4+ T cells were significantly reduced in patients with SLE (Zhu et al., 2019). Several recent studies (Talaat et al., 2015; Mizui and Tsokos, 2018) have confirmed that Treg cells in SLE patients exhibit both reduced numbers and functional defects compared to those in healthy individuals. We believe that increasing the quantity or enhancing the function of Treg cells may be the key to remission in patients with severe SLE.

There are several limitations to this study that should be acknowledged. Firstly, the sample size was relatively small, which may limit the generalizability of our findings. Additionally, although we identified AMPH as a hub gene associated with SLE severity, we did not perform further functional experiments to validate its role in the disease. In conclusion, our study suggests that multiorgan functional impairment and upregulation of AMPH may contribute to decreased proportion of Treg cells, which in turn play a critical role in the severity of SLE. Therefore, targeting Treg cells may represent a promising therapeutic strategy for patients with severe SLE. However, further studies with larger sample sizes and functional experiments are needed to confirm our findings and elucidate the underlying mechanisms of SLE pathogenesis.

## Data Availability

The scRNA-seq datasets used in this study is publicly available on the Gene Expression Omnibus database with the accession number GSE135779. The original contributions presented in the study are publicly available. This data can be found here: https://www.ncbi.nlm.nih.gov/geo/query/acc.cgi?acc=GSE228066. Further inquiries can be directed to the corresponding authors.
